# Effect of pregnane X receptor expression on drug resistance in breast cancer

**DOI:** 10.3892/ol.2014.1817

**Published:** 2014-01-22

**Authors:** EN-QI QIAO, HONG-JIAN YANG

**Affiliations:** Department of Oncological Surgery, Zhejiang Cancer Hospital, Hangzhou, Zhejiang 310022, P.R. China

**Keywords:** pregnane X receptor, breast carcinoma, SR12813, drug resistance, apoptosis

## Abstract

This study was conducted to investigate the effect of increased expression of the nuclear transcription factor receptor pregnane X receptor (PXR) on drug resistance of breast cancer cells. Western blotting was used to detect the expression of PXR in breast carcinoma cells. The PXR agonist SR12813 was used to upregulate the expression of PXR. Semi-quantitative polymerase chain reaction was used to detect PXR gene expression in normal and cancerous breast tissues, as well as the expression levels of the drug-resistant genes multidrug resistance protein 1 (MDR1) and breast cancer resistance protein (BCRP) in breast cancer cells. A Cell Counting Kit-8 assay was used to observe the sensitivity of the breast cancer cells to chemotherapeutic agents. Flow cytometry was used to investigate cell apoptosis. PXR expression was detected in normal and cancerous human breast tissues and in breast cancer cell lines. SR12813 treatment led to an increased expression of PXR protein and an increased expression of drug-resistant genes, MDR1 and BCRP, in MCF-7 and MDA-MB-231 cells. SR12813 pretreatment significantly increased the resistance of MDA-MB-231 cells to docetaxel. A marked increase in resistance to 4-hydroxytamoxifen was also observed in MCF-7 with SR12813 pretreatment. Additionally, we also found that pretreatment with SR12813 led to reduced apoptosis of the two cell strains induced by chemotherapeutic agents. In conclusion, PXR expression has an important effect on the sensitivity to chemotherapy of PXR-positive breast carcinoma. The inhibitory effect of PXR on cell apoptosis may contribute to the drug resistance of breast carcinoma.

## Introduction

Breast cancer is the most common malignancy and the second leading cause of cancer-related mortality among females worldwide (1). Chemotherapy is one of the most important therapeutic approaches for breast cancer patients; however, the efficacies of drug treatments on breast cancers are often limited due to the resistance of tumor cells (2).

The pregnane X receptor (PXR) belongs to the nuclear hormone receptor (NR) superfamily of ligand-activated transcription factors (3), and is alternatively referred to as the steroid and xenobiotic receptor (SXR) or the pregnane-activated receptor (PAR), also termed as PXR or hPXR in humans. PXR regulates the expression of a number of downstream targeted genes, which are mostly related to the metabolism and transport of xenobiotics and associated with drug resistance in a number of cancers (4), such as cytochrome P450 (CYP450), multidrug resistance 1 (MDR1), breast cancer resistance protein (BCRP) and multidrug resistance-associated protein 2 (5–7).

In this study, we used SR12813, a potent and selective agonist of hPXR, to upregulate and activate the PXR protein in breast cancer cells, and analyzed the correlation between PXR and drug resistance in breast cancer, this study was designed to explore the formation mechanism of drug resistance of breast cancer cells and provide theoretical basis for clinical chemotherapy.

## Materials and methods

### Materials

Thirty-three breast carcinoma tissues and corresponding normal tissues were obtained from Jiangsu Cancer Hospital Affiliated to Nanjing Medical University (Nanjing, China). Informed consent was provided in compliance with the Declaration of Helsinki. Breast cancer cell lines, MCF-7 and MDA-MB-231, were purchased from Cell Bank of the Chinese Academy of Sciences, Shanghai Life Science Institute (Shanghai, China). Cells were cultured in Dulbecco’s modified Eagle’s medium (DMEM)-high glucose (Invitrogen, Carlsbad, CA, USA) supplemented with 10% fetal bovine serum (Invitrogen) and 100 U/ml penicillin-streptomycin (Invitrogen).

### Reagents and instruments

hPXR (H-11) sc-48340 mouse monoclonal antibody and relevant horseradish peroxidase (HRP)-labeled secondary antibodies were purchased from Santa Cruz Biotechnology Inc., (Santa Cruz, CA, USA). β-actin AP0060 rabbit antibody was from Bioworld Technology, Inc. (Minneapolis, MN, USA). SR12813, dissolved in dimethyl sulfoxide (DMSO), and 4-hydroxytamoxifen were purchased from Sigma-Aldrich (St. Louis, MO, USA). Docetaxel was purchased from Qilu Pharmaceutical Co., Ltd (Jinan, China). The Cell Counting Kit-8 (CCK-8) cell proliferation-toxicity test kits were from Dojindo (Kumamoto, Japan). An RNA extraction kit, reverse transcription system and semi-quantitative PCR reagents were purchased from Takara Bio Inc. (Shiga, Japan). The total protein extraction kit was purchased from Beyotime Institute of Biotechnology (Shanghai, China). The enhanced chemiluminescence (ECL) detection system was purchased from Millipore (Billerica, MA, USA). The 7300 Real Time PCR system was from ABI (Warrington, UK). The Microplate Reader system was from Promega (Madison, WI, USA) and the BD FACSCalibur Flow Cytometer was purchased from Becton-Dickinson (New York, NY, USA).

### Western blotting

Cells were directly lysed with 2X sodium dodecyl sulfate-polyacrylamide gel electrophoresis (SDS-PAGE) sample buffer (Beyotime Institute of Biotechnology), then boiled and sonicated. The total protein was obtained following centrifugation for 10 min at 15,407 × g, then total protein was isolated, separated on a 10% SDS-PAGE gel and transferred to polyvinylidene difluoride (PVDF) membranes at 100 V for 1 h. Then, the PVDF membranes were incubated with either anti-hPXR monoclonal antibody H-11 (diluted to 1:800 in blocking buffer) or anti-β-actin antibody AP0060 (diluted to 1:4000 in blocking buffer) overnight at 4°C. For staining, a goat anti-mouse HRP-labeled secondary antibody (diluted to 1:10,000 in blocking buffer) or goat anti-rabbit HRP-labeled secondary antibody (diluted to 1:2,000 in blocking buffer) were used for 1.5 h at room temperature. The protein bands were detected by an ECL detection system. Following normalization by the corresponding expression of β-actin, protein expression levels of PXR were determined by densitometry scans.

### Semi-quantitative RT-PCR

Total RNA was extracted from cell cultures and from breast cancer and corresponding normal tissues with TRIzol^®^ reagent (Invitrogen). Reverse transcription was performed by a Superscript T3000 Thermocycler system (Mettler-Toledo, Biometra Goettingen, Giessen, Germany). SYBR Green-based semi-quantitative PCR was used to measure relative gene expression with the following primer pairs: hPXR: 5′-CGA GCT CCG CAG CAT CA-3′ and 5′-TGT ATG TCC TGG ATG CGC A-3′; MDR1: 5′-GTT GCT GCT TAC ATT CAG GTT TC-3′ and 5′-ACC AGC CTA TCT CCT GTC GC-3′; BCRP: 5′-TCC ACT GCT GTG GCA TTA AA-3′ and 5′-TGC TGA AAC ACT GGT TGG TC-3′; β-actin: 5′-TCA CCC ACA CTG TGC CCA TCT ACG A-3′ and 5′-CAG CGG AAC CGC TCA TTG CCA ATG G-3′. PCR conditions were as follows: One cycle at 95°C for 30 sec, followed by 40 cycles of PCR amplification, each consisting of 95°C for 5 sec and 60°C for 31 sec. The concentration of mRNA was calculated according to the standard curve and then normalized to that of β-actin. The data processing methods were according to a previous study (8).

### CCK-8 assay

The cells were seeded in 96-well plates at an initial density of 8,000 cells per well. After incubation for 12 h, cells were treated with 4-hydroxytamoxifen or docetaxel at different concentration gradients (4-hydroxytamoxifen, 0.5, 1.0, 5.0, 10.0, 15.0 and 20.0 μM; docetaxcel, 0.05, 0.1, 0.2, 0.4, 0.8 and 1.6 μg/ml) for 24, 48 or 72 h directly or following a 24 h treatment of 0.3 μM SR12813 or 0.1% DMSO. For the evaluation of SR12813 cell cytotoxicity, cells were treated with SR12813 (0.3 μM) or 0.1% DMSO for 24, 48 or 72 h. The cell viability was measured by CCK-8 assay according to the manufacturer’s instructions. In brief, 90 μl fresh serum-free medium and 10 μl CCK-8 reagent were added into each well after decanting the old medium and culture was continued at 37°C for 1 h. The optical density (OD) at 450 nm was measured by a microplate reader (Promega). The above steps were repeated three times and the average was calculated. The cell viability fraction (%) was calculated as follows: [OD_450 nm in test cells_ − OD_450 nm in blank control_]/[OD_450 nm in control cells_ − OD_450 nm in blank control_]. The relative drug resistance folds were analyzed by comparison with IC_50_.

### Flow cytometry assay of cell apoptosis

The MCF-7 and MDA-MB-231 cells were inoculated in six-well plates at a density of 2×10^5^ cells/well. They were respectively divided into three groups, including a control group, single drug group and SR12813 (0.3 μM) pretreatment group, and each group was set into three repeated wells. After 24 h, the cells were treated respectively with complete medium containing 5 μM 4-hydroxytamoxifen (MCF-7) or 0.1 μg/ml docetaxel (MDA-MB-231) for 24 h. The control group cells were treated with complete medium containing the same volume of phosphate-buffered saline (PBS). Cell apoptosis was detected by BD FACSCalibur flow cytometry. The cells were detached with 0.25% trypsin and resuspended in PBS. Fluorescein isothiocyanate (FITC)-Annexin V and propidium iodide (PI) were added to 10^5^ cells, after which the cells were placed in the dark at room temperature for 15 min according to the manufacturer’s instructions (Apoptosis Detection kit; BD Biosciences, Franklin Lakes, NJ, USA). Early apoptotic cells were stained with FITC-Annexin V (20 μg/ml) alone, and late apoptotic cells and necrotic cells were stained with FITC-Annexin V and PI (50 μg/ml).

### Statistical analysis

Statistical analysis was performed using SPSS 16.0 software (SPSS Inc., Chicago, IL, USA). Data are expressed as the mean ± standard deviation. Student’s t-test (two-tailed) was used to analyze the difference between two groups and one-way analysis of variance was used to analyze the difference among three groups. Data were considered to be statistically significant when P≤0.05.

## Results

### Comparison of PXR gene expressions between in breast carcinoma tissues and corresponding normal tissues

Comparison of PXR gene expression levels between breast carcinoma tissues and corresponding normal tissues is shown in Table I. The data showed that there is higher expression of PXR in breast cancer tissues. Compared with the normal breast tissues, the expression levels of PXR gene were significantly higher in breast carcinoma tissues (t=17.979, P<0.001), ~6.92±1.86 times according to the statistical results.

### Increased PXR protein expression levels in MCF-7 and MDA-MB-231 cells following SR12813 treatment

**Figure 1:**
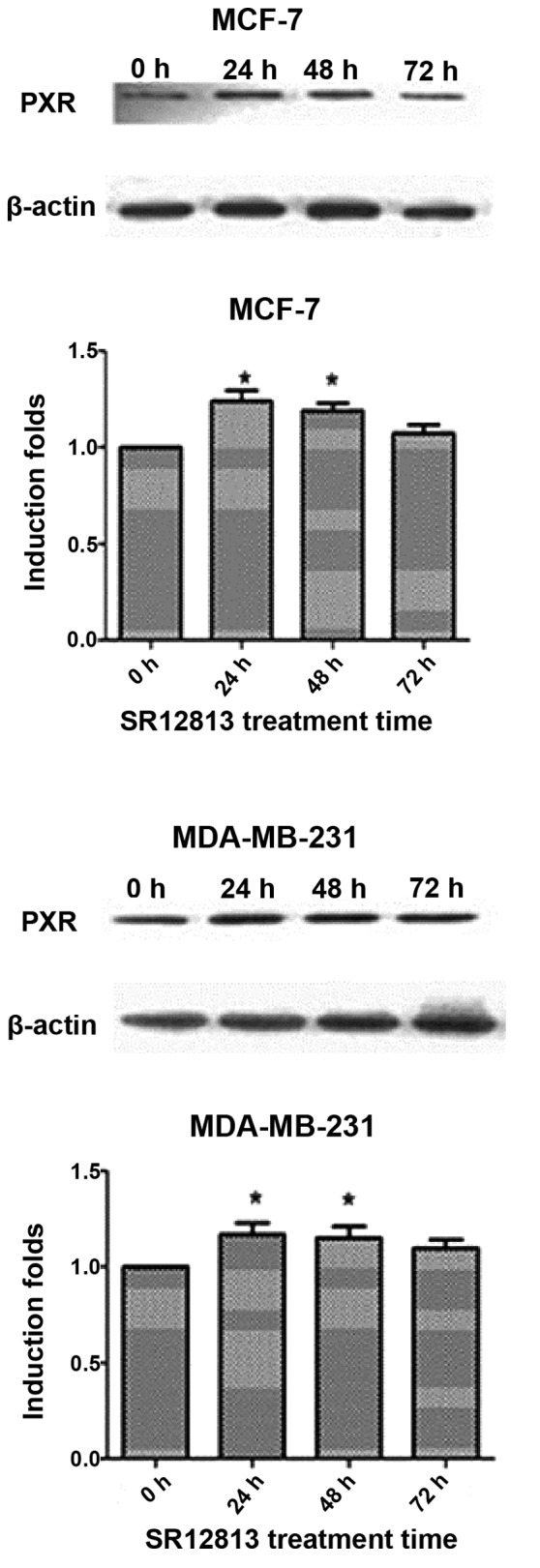
Changes of PXR protein levels in breast cancer cells following SR12813 treatment. After 0.3 μM SR12813 treatment for 0, 24, 48 or 72 h, the PXR protein levels were evaluated by western blot assay and normalized by β-actin. Data are the mean results from three independent experiments. ^*^P<0.05, compared with the control group (0 h group). PXR, pregnane X receptor.

MDA-MB-231 cells had a higher PXR expression compared with MCF-7. After treatment with 0.3 μM SR12813 for 24, 48 or 72 h, the levels of PXR protein were increased (particularly at 24 and 48 h) (P<0.05) (Fig. 1).

### Changes in MDR1 and BCRP gene expression levels in breast cancer cells before and after SR12813 treatment

**Figure 2:**
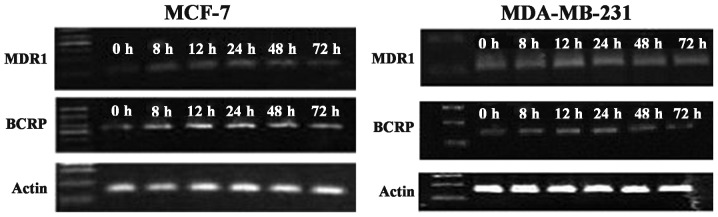
Activated pregnane X receptor enhances MDR1 and BCRP gene levels in MCF-7 and MDA-MB-231 cells. After treatment with 0.3 μM SR12813 for 0, 8, 12, 24, 48 or 72 h, the MDR1 and BCRP gene levels were evaluated by SYBR Green-based semi-quantitative polymerase chain reaction assay and normalized by β-actin. MDR1, multidrug resistance protein 1; BCRP, breast cancer resistance protein.

Compared with the control group, after treatment with 0.3 μM SR12813 for 8, 12, 24, 48 and 72 h, the levels of MDR1 and BCRP mRNA in MCF-7 or MDA-MB-231 cells were significantly increased by 1.5–4 times. The increased levels of MDR1 and BCRP mRNA in MCF-7 treated by SR12813 at 24 h were highest, 3.61±0.33 and 3.24±0.17 times higher, respectively. The levels of MDR1 and BCRP mRNA in breast cancer cell lines MCF-7 or MDA-MB-231 before to and following SR12813 treatment were significantly different (P<0.001). In MDA-MB-231 cells treated with 0.3 μM SR12813, the levels of MDR1 mRNA were highest at 12 h (up to 3.61±0.25 times; P<0.001). BCRP mRNA levels were highest at 12 and 24 h (3.28±0.24 and 3.23±0.16 times, respectively; P<0.001) (Fig. 2). Combined with the western blot assay, the results indicate that activated PXR enhances MDR1 and BCRP gene levels in breast cancer cells.

### Increased resistance of MCF-7 and MDA-MB-231 to therapeutic agents after upregulated PXR expression by SR12813 treatment

The IC_50_ values of 4-hydroxytamoxifen to MCF-7 cells at 48 h were 9.81±0.49 μM in the control group, 9.40±0.69 μM in the DMSO treatment group and 11.57±0.83 μM in the SR12813 pretreatment group. At 72 h, these values were 8.35±0.64, 8.22±0.59 and 9.78±0.68 μM, respectively. The differences between the SR12813 pretreatment group and the other groups were significant (P<0.05) (Table II). Similarly, the IC_50_ values of docetaxel to MDA-MB-231 cells at 24 h were 0.45±0.025 μg/ml in the control group, 0.44±0.021 μg/ml in the DMSO treatment group and 0.67±0.091 μg/ml in the SR12813 pretreatment group. At 48 h, these values were 0.40±0.042, 0.39±0.025 and 0.53±0.056 μg/ml, and at 72 h, these values were 0.35±0.021, 0.36±0.036 and 0.46±0.040 μg/ml, respectively. These values in the SR12813 pretreatment group were significantly different compared with the other groups at 24 h (P=0.003), 48 h (P=0.015) and 72 h (P=0.025) (Table III).

### Effect of PXR on MCF-7 and MDA-MB-231 cell apoptosis induced by therapeutic agents

**Figure 3:**
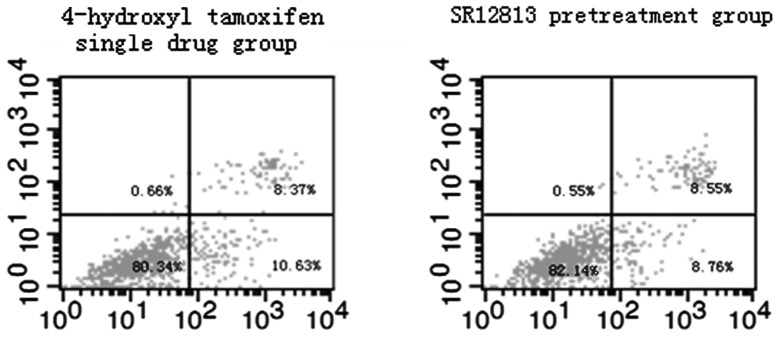
Results of MCF-7 apoptosis. The total rate of apoptosis in one-time independent experiment was 5.79% in the control group, 19.00% in the 4-hydroxyltamoxifen single drug group and 17.15% in the SR12813 pretreatment group.

**Figure 4:**
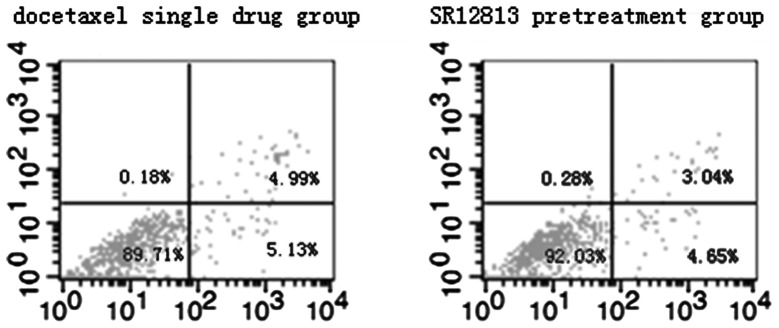
Results of MDA-MB-231 apoptosis. The total rate of apoptosis in a one-time independent experiment was 5.17% in the control group, 10.12% in the docetaxel single drug group and 7.69% in the SR12813 pretreatment group.

Results of flow cytometric assays from control and experimental treatment groups are shown in Figs. 3 and 4. The total rates of apoptosis of cells in the control group were 5.81±0.62% (MCF-7) and 5.17±0.46% (MDA-MB-231), which were significantly increased after treatment with therapeutic agents for 24 h (P<0.001). Compared with the single drug groups (19.43±0.97 and 10.27±0.80%, in MCF-7 and MDA-MB-231 cells, respectively), the total rates of apoptosis of cells in the SR12813 pretreatment groups were significantly decreased (17.26±0.40%, t=3.571, P=0.023 and 7.69±0.54%, t=4.647, P=0.01 in MCF-7 and MDA-MB-231 cells, respectively).

## Discussion

Drug resistance of tumor cells is a complicated process with multiple factors, genes and steps. PXR is a member of the ligand-activated transcription factor superfamily called nuclear receptor subfamily 1 group I member 2 (NR1I2) whose downstream target genes are involved in the production of phase I metabolic enzymes, including CYP450 enzymes, phase II metabolic enzymes, including glucuronyltransferase (UGT) and phase III drug transporters, including P-glycoprotein (P-gp) and BCRP (6,7). Since these target genes are mostly involved in the formation of tumor MDR, PXR may be a novel master regulator of drug resistance.

P-gp and BCRP are the main members of the ATP binding cassette (ABC) superfamily of transporters, and are important PXR target genes (6). Their normal physiologic function is to protect the body from cytotoxicity caused by drugs or other xenobiotics. P-gp overexpression is a major cause of MDR. The protein is able to increase the outflow of a number of intracellular structurally and functionally unrelated chemotherapy drugs, including anthracyclines and taxanes, thereby reducing the efficacy (9). Navarro *et al* (10) reported that the combination of adriamycin and P-gp silencing formulations led to a double increase of adriamycin uptake and a significant improvement of the therapeutic effect of adriamycin in MCF-7 cells. Yuan *et al* (11) demonstrated there was a significant negative correlation between BCRP expression and 5-FU resistance in breast cancer cells, which may be used to optimize the chemotherapy scheme in BCRP-positive breast cancer patients. Liu *et al* (12) also found that P-gp and BCRP proteins are implicated in the formation of MDR in breast cancer and P-gp expression can be used to evaluate the efficacy of anthracycline chemotherapy in breast cancer. It is thus clear that the two proteins play an important role in breast cancer resistance mechanisms. Our study results demonstrated that treatment with SR12813 resulted in an upregulation of MDR1 and BCRP gene levels. Correspondingly, there was a significant increase in breast cancer cell resistance, which was consistent with the above findings.

A number of studies have demonstrated that PXR is involved in MDR of tumor cells. Chen *et al* (13) demonstrated that PXR is expressed in normal and cancerous prostate tissues and in the prostate cancer cell line PC-3. Treatment with SR12813 activated PXR and improved the level of MDR1, and increased the resistance of PC-3 cells to paclitaxel or vinblastine. The targeted knockdown of PXR reduced the resistance of PC-3 cells to paclitaxel or vinblastine. Similarly, Raynal *et al* (14) reported that activation of PXR increased the resistance of colorectal cancer cells to irinotecan. The increase in chemoresistance was reversed by the PXR antagonist sulforaphane. For breast cancer, Dotzlaw *et al* (15) first detected the expression of PXR mRNA in human breast cancer tissues by PCR amplification. Conde *et al* (16) further demonstrated the overexpression of PXR protein in breast cancer cells by immunohistochemistry and western blot analysis. The organic anion transporter polypeptide 1A2 (OATP1A2) is capable of mediating the cellular uptake of estrogen metabolites. Meyer zu Schwabedissen *et al* (17) identified that the PXR-OATP1A2 promoter interaction induced the expression of OATP1A2 in breast cancer cells, increased the uptake of the estrogen metabolite estrone 3-sulfate, and improved the estrogen effect of breast cancer. This process was also reversed by a specific antagonist of PXR, A-792611. Chen *et al* (18) confirmed hPXR expression in normal and cancerous human breast specimens and in breast cancer cell lines MCF-7 and MDA-MB-231. Activation of hPXR by SR12813 led to an increased expression of CYP3A4 and MDR1 in the two cell strains and improved resistance to Taxol or tamoxifen. Furthermore, knockdown of hPXR via shRNA sensitized the two cell strains to Taxol, vinblastine or tamoxifen. Jiang *et al* (19) demonstrated that PXR mRNA and protein were expressed in human colon cancer cell lines. The expression of PXR was increased following treatment with the PXR agonist rifampicin. PXR being activated or knocked down, accordingly increased or inhibited the cell proliferation, and enhanced or reduced the resistance of cells to the chemotherapeutic agents. However, the specific mechanism is unknown. This is one of the limited studies on PXR and tumor resistance. Our results demonstrated that treatment with SR12813 increased hPXR expression and significantly increased the expression of MDR1 and BCRP in MDA-MB-231 and MCF-7 cells, and distinctly reduced sensitivity to 4-hydroxytamoxifen or docetaxel, which was mostly consistent with previous studies. However, there were also some opposing study results. Honorat *et al* (20) found that PXR was also able to downregulate ABCG2 expression in PXR- and glucocorticoid receptor-positive MCF-7 cells and improved the sensitivity of MCF-7 to mitoxantrone.

Studies have demonstrated that PXR is implicated in apoptosis of tumor cells, which may be an important cause of MDR. Masuyama *et al* (21) confirmed that PXR overexpression led to a significant decrease in endometrial cancer cell growth inhibition and inhibited apoptosis induced by cisplatin or paclitaxel. Wang *et al* (22) demonstrated that in colon cancer cells, activated PXR was able to induce fibroblast growth factor 19 (FGF19)-dependent cancer cell proliferation and inhibit cell apoptosis. Moreover, the authors observed that PXR caused FGF19 activation only in the cancer cells. The present study also demonstrated that following activation and upregulated PXR expression by SR12813 treatment in MCF-7 and MDA-MB-231 cells, cell apoptosis induced by 4-hydroxytamoxifen or docetaxel was significantly inhibited. The data were consistent with the above previous findings; however, the specific mechanism requires further exploration. Constrastingly, Liu *et al* (23) demonstrated that Tanshinone IIA (Tan IIA) had marked growth inhibition effects on U-937 cells through the induction of cell apoptosis. The Tan IIA-induced apoptosis may be due to the activation of PXR, which inhibited the activity of NF-κB and led to the downregulation of monocyte chemoattractant protein (MCP)-1 (MCP-1/CCL2) expression. The aforementioned results indicate that PXR plays a dual role in tumor cell apoptosis.

To date, literature that discusses the correlation between PXR and breast cancer are relatively limited and the majority are related to colon cancer. In our study, we detected the expression of PXR in normal and cancerous breast tissues and in breast cancer cell lines. PXR played a significant role in MDR of breast cancer cells, and treatment with the PXR agonist SR12813 activated and increased PXR protein expression, increased the resistance of cancer cells to chemotherapy drugs and decreased cell apoptosis. The specific mechanisms and the correlation between PXR and the development of breast cancer are yet to be explored. There are a number of opposing results among the studies discussed; therefore, further investigation is required.

## Figures and Tables

**Table I:** Comparison of PXR gene expression between in breast carcinoma tissues and corresponding normal tissues.

Group	n	^Δ^CT	2^−ΔΔCT^	T-value	P-value
T	33	3.34±0.51			
N	33	6.10±0.72	6.92±1.86	17.979	<0.001

Student’s t-test (two-tailed) was used to analyze the difference between the two groups. Data are presented as the mean ± standard deviation. T, tumor tissue; N, normal breast tissue; PXR, pregnane X receptor.

**Table II:** Effect of PXR on MCF-7 cell sensitivity to 4-hydroxytamoxifen.

Group	IC_50_ at 48 h (μM)	F-value	P-value	IC_50_ at 72 h (μM)	F-value	P-value
Control	9.81±0.49			8.35±0.64		
DMSO treatment	9.40±0.69			8.22±0.59		
SR12813 pretreatment	11.57±0.83^a^	8.529	0.018	9.78±0.68^a^	16.295	0.045

MCF-7 cells were seeded in 96-well plates at an initial density of 8,000 cells per well. After incubation for 12 h, cells were treated with 4-hydroxytamoxifen at different concentration gradients for 24, 48 or 72 h directly or following a 24 h treatment of 0.3 μM SR12813 or 0.1% DMSO. The viability percentages of cells with no therapeutic agents were deemed as 100%.

a P<0.05 and

b P<0.01, assayed by one-way analysis of variance, compared with the control group or DMSO treatment group.

The results are the average from three separate experiments and presented as mean ± standard deviation. PXR, pregnane X receptor; DMSO, dimethyl sulfoxide.

**Table III:** Effect of PXR on MDA-MB-231 cell sensitivity to docetaxel.

Group	IC_50_ at 24 h (μg/ml)	F-value	P-value	IC_50_ at 48 h (μg/ml)	F-value	P-value	IC_50_ at 72 h (μg/ml)	T-value	P-value
Control	0.45±0.025			0.40±0.042			0.35±0.021		
DMSO treatment	0.44±0.021			0.39±0.025			0.36±0.036		
SR12813 pretreatment	0.67±0.091^b^	16.885	0.003	0.53±0.056^a^	9.079	0.015	0.46±0.040^a^	7.248	0.025

MDA-MB-231 cells were seeded in 96-well plates at an initial density of 8,000 cells per well. After incubation for 12 h, cells were treated with docetaxel at different concentration gradients for 24, 48 or 72 h directly or following a 24 h treatment of 0.3 μM SR12813 or 0.1% DMSO. The viability percentages of cells with no therapeutic agents were deemed as 100%.

a P<0.05 and

b P<0.01, assayed by one-way analysis of variance, compared with the control group or DMSO treatment group.

Results are the average from three separate experiments and presented as mean ± standard deviation. PXR, pregnane X receptor; DMSO, dimethyl sulfoxide.
